# Improving the Design of MEMS INS-Aided PLLs for GNSS Carrier Phase Measurement under High Dynamics

**DOI:** 10.3390/mi8050135

**Published:** 2017-04-25

**Authors:** Tisheng Zhang, Yalong Ban, Xiaoji Niu, Wenfei Guo, Jingnan Liu

**Affiliations:** GNSS Research Center, Wuhan University, 129 Luoyu Road, Wuhan 430079, China; zts@whu.edu.cn (T.Z.); ylb@whu.edu.cn (Y.B.); wf.guo@whu.edu.cn (W.G.); jnliu@whu.edu.cn (J.L.)

**Keywords:** INS-aided PLLs, carrier phase, tracking error, high dynamics, GNSS/INS deeply-coupled system, MEMS INS

## Abstract

The phase locked loop (PLL) bandwidth suffers a dilemma on carrier phase accuracy and dynamic stress tolerance in stand-alone global navigation satellite systems (GNSS) receivers. With inertial navigation system (INS) aiding, PLLs only need to tolerate aiding information error, instead of dynamic stress. To obtain accurate carrier phase under high dynamics, INS-aided PLLs need be optimally designed to reduce the impact of aiding information error. Typical micro-electro-mechanical systems (MEMS) INS-aided PLLs are implemented and tested under high dynamics. Tests using simulation show there is a step change in the aiding information at each integer second, which deteriorates the carrier phase accuracy. An improved structure of INS-aided PLLs is proposed to eliminate the step change impact. Even when the jerk is 2000 m/s^3^, the tracking error of the proposed INS-aided PLL is no more than 3°. Finally, the performances of stand-alone PLLs and INS-aided PLLs are compared using field tests. When the antenna jerk is 300 m/s^3^, the carrier phase error from the stand-alone PLLs significantly increased, while the carrier phase error from the MEMS INS-aided PLLs almost remained the same. Therefore, the proposed INS-aided PLLs can suppress tracking errors caused by noise and dynamic stress simultaneously under high dynamics.

## 1. Introduction

With the development of global navigation satellite systems (GNSS), precision positioning is gradually being applied in dynamic environments, including mobile mapping, autonomous driving, seismic surveying, precision weapon guidance, and other areas [1,2,3,4]. GNSS carrier phases, observations of precision positioning, are measured by phase locked loops (PLLs). The PLL bandwidth design suffers a dilemma with accuracy and dynamic stress tolerance [5]. To reduce the noise and improve accuracy, the tracking bandwidth should be narrow [6]. However, to be better suited for dynamic stress, a wider tracking bandwidth is ideal [7,8]. Therefore, in a stand-alone GNSS receiver, a trade-off design of PLL bandwidth is required to adapt various scenarios [9]. The carrier phase accuracy is worsened in dynamic environments.

External sensor aiding is considered as an alternative to improve carrier phase tracking performance. The inertial navigation system (INS) has a superior dynamic characteristic, which is highly complementary to GNSS. Therefore, GNSS/INS integration is widely studied and applied [10,11,12,13,14]. When the INS is used to assist the GNSS receiver baseband, the structure is named deeply coupled system [15,16,17]. The INS information assists PLLs based on the concept of feed-forward [18,19]. In INS-aided PLLs, with the aiding of the INS feed-forward branch, PLLs only need to tolerate the error from the aiding information, instead of vehicle dynamic stress. Therefore, INS-aided PLLs do not need to increase loop bandwidth to tolerate dynamics, and the carrier phase tracking performance is better than that of stand-alone PLLs [20,21].

The concept of INS-aided PLLs has been proposed and studied in the GNSS community. Alban et al. proposed the INS-aided tracking loop architecture, but its advantage was not fully shown due to simple implementation [18]. Chiou et al. tested the sensitivity of INS-aided PLLs, with the same design as Alban’s [21]. Mike et al. adjusted the aiding information update rate by extrapolation of the INS velocity, and realized the carrier phase tracking under an acceleration of 40G [4]. However, INS-aided PLLs were not optimized to improve carrier phase accuracy. Tsujii et al. tested the INS-aided GPS tracking performance under strong ionospheric scintillations, without purposeful design for high dynamics [17]. Therefore, the tracking error caused by aiding information needed to be analyzed in detail, and INS-aided PLLs needs to be optimally designed to improve the carrier phase accuracy under high dynamics.

To obtain accurate GNSS carrier phase under dynamics, we focus on improving the design of INS-aided PLLs. First, micro-electro-mechanical systems (MEMS) INS-aided PLLs were implemented in our software GNSS/INS deeply-coupled system, and tracking errors caused by aiding information were tested and analyzed under a jerk of 2000 m/s^3^ using hardware simulator. Then, INS-aided PLLs were improved to reduce tracking errors caused by aiding information. Finally, the performance of improved MEMS INS-aided PLLs was evaluated under a jerk of 300 m/s^3^ through field tests.

## 2. Tracking Error Analysis of INS-Aided PLLs

To analyze tracking errors, MEMS INS-aided PLLs were introduced, implemented in our software GNSS/INS deeply-coupled system. Then, INS-aided PLLs were tested using GPS/INS hardware simulator, and tracking errors caused by aiding information were analyzed and used to guide INS-aided PLLs optimization.

### 2.1. Implementation of INS-Aided PLLs

**Figure 1:**
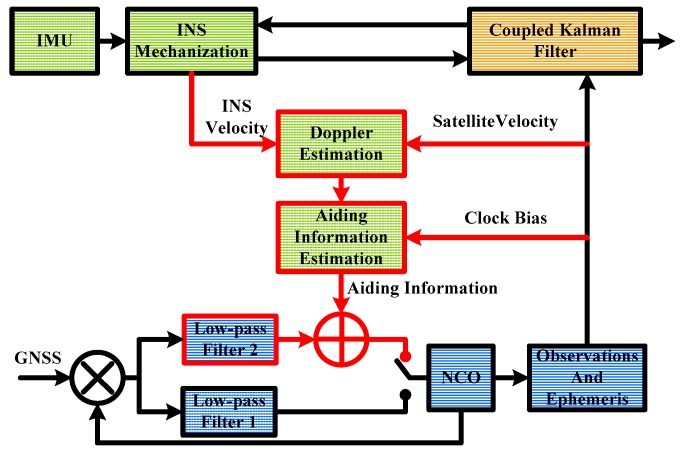
Structure of inertial navigation system (INS) aided phase locked loop (PLL) implementation.

The structure of INS-aided PLLs implemented in the GNSS/INS deeply-coupled system is shown in Figure 1. The lower part is the tracking loop branch. When the discriminator output was sent to the low-pass filter 1, it worked as a normal PLL. When the discriminator output was sent to the low-pass filter 2, it worked as an INS-aided PLL. Once the frame synchronization was achieved, the observations and satellite ephemeris could be obtained. The upper part is the INS branch. The inertial measurement unit (IMU) includes three-axis gyroscopes and three-axis accelerometers. The INS mechanization was set to output position, velocity, and attitude. The Kalman filter was to couple GNSS and INS, to restrain divergence of navigation results.

The Doppler measured by the low-pass filter 1 consisted of Doppler between receiver and satellite, frequency error of the receiver clock, and thermal noise. The Doppler between receiver and satellite could be estimated by the INS and satellite ephemeris. When we calculated the receiver velocity, we could also obtain the receiver clock bias. Therefore, with the aiding information assist, the low-pass filter 2 only needed to track thermal noise and the aiding information error.

The estimated Doppler can be expressed as the velocity of the receiver relative to the satellite, projected onto the line-of-sight (LOS) vector. The relationship is expressed in Equation (1) [22]:

(1) $$f_{\mathrm{dopp},k}=e_{k}^{T}\left(\frac{V_{\mathrm{INS}}-V_{\mathrm{SV},k}}{\lambda }\right)$$

where, $e_{k}^{T}$ is unit LOS vector from receiver to satellite *k*, *V*_INS_ is velocity of INS, which is the same as receiver, *V*_SV,*k*_ is velocity of satellite *k*, and λ is carrier wavelength.

The estimated Doppler update rate and the numerically controlled oscillator (NCO) update rate may be different and asynchronous. Therefore, the estimated Doppler needed to be extrapolated to the PLL maximum update rate. Because the satellite acceleration is a constant in a short time period, precise Doppler could be obtained via linear extrapolation. The *n*-th estimated Doppler via extrapolation is expressed in Equation (2) [23]:

(2) $$f_{\mathrm{dopp},k,n}=f_{\mathrm{dopp},k}+e_{k}^{T}(\frac{a_{\mathrm{INS}}}{\lambda }+\frac{V_{\mathrm{SV},k,i}-V_{\mathrm{SV},k,i-1}}{\lambda })\frac{n}{1000}$$

where, *a*_INS_ is acceleration of INS, *V*_SV,*k*,*i*_ is velocity of satellite *k* at the *i*-th second, *n* is the number of extrapolation.

The receiver works with normal PLLs before the aiding information is ready. When the difference between the aiding information and the low-pass filter 1 remained small enough, INS-aided PLLs worked as expected. Since the clock drift was smaller than the noise of the calculated clock bias, the clock drift was tracked by the low-pass filter 2. In addition, the acceleration of satellite was no more than 0.2 m/s^2^. Therefore, the estimated error of the aiding information was mainly from the INS velocity.

### 2.2. Tracking Error Analysis

**Figure 2:**
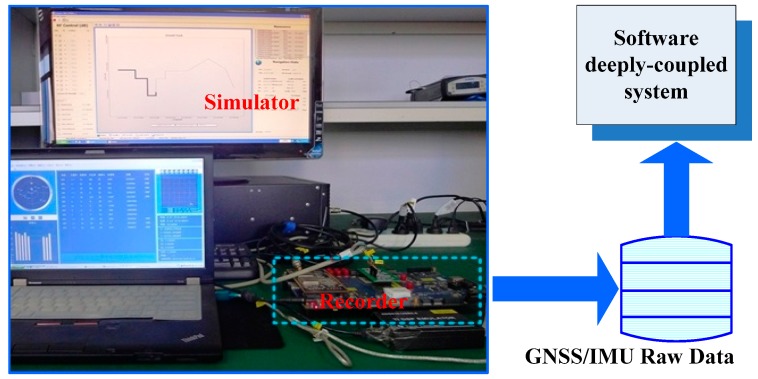
Tracking error testing set.

**Figure 3:**
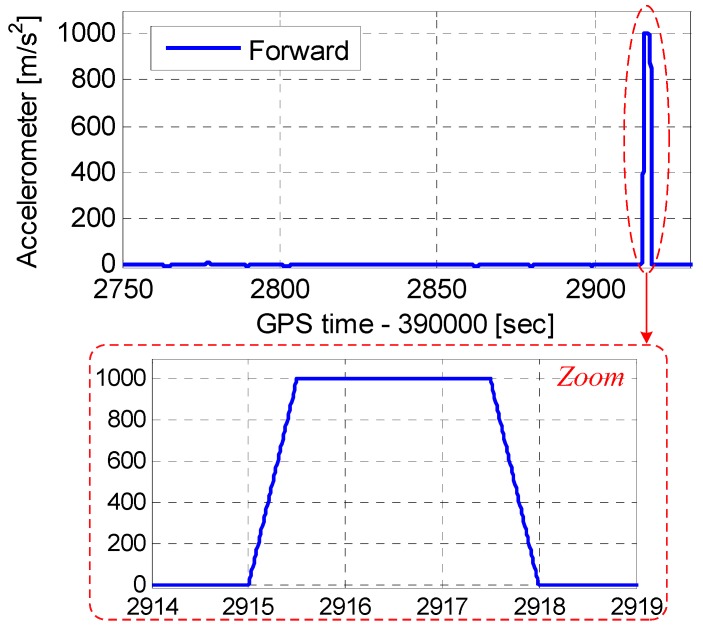
Vehicle dynamics in the simulation scenario.

The tracking error testing set is shown in Figure 2. To test tracking errors quantitatively, a GPS/INS hardware simulator was used. A GPS/IMU data synchronous recorder, developed by ourselves, was connected to the simulator to sample GPS intermediate frequency (IF) data and IMU raw data. The raw data was processed using our GNSS/INS deeply-coupled system. Since the tracking errors caused by thermal noise and clock were analyzed in literatures, we focused on analyzing the impact of the aiding information on tracking error [24]. To highlight the tracking error caused by aiding information, the impact of thermal noise and clock should be reduced as much as possible. Hence, an oven controlled crystal oscillator (OCXO) was used in the recorder to reduce the clock impact on the tracking error, and the carrier to noise ratio (CNR) of the GPS signal was set to 100 dB-Hz, to minimize the thermal noise effect. The IMU parameters set in the simulator were based on a typical MEMS IMU STIM300 [25]. Figure 3 shows the vehicle dynamics in the motion scenario. The accelerations were set to less than 10 m/s^2^ before 392,915 s. The dynamic had an acceleration of 1000 m/s^2^, and a jerk of 2000 m/s^3^ was set at 392,915–39,298 s.

**Figure 4:**
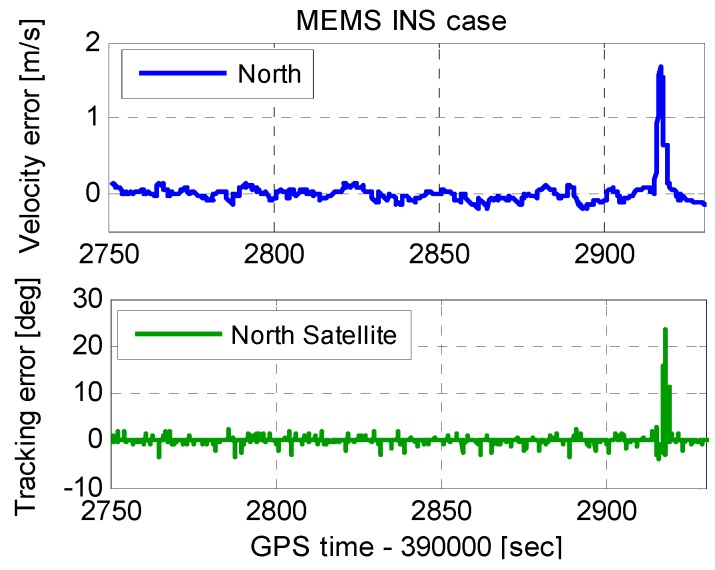
INS velocity error and PLL tracking error.

To tolerate high dynamics, the INS-aided PLLs with a bandwidth of 20 Hz, and an integration time of 1 ms, was used. Figure 4 shows the INS velocity error and PLL tracking error. When the vehicle acceleration in the scenario was less than 10 m/s^2^, the INS velocity error affected by dynamics was not obvious. However, when the vehicle acceleration was 1000 m/s^2^, the INS velocity error was approximately 1.7 m/s. Since the velocity error enters PLLs with the aiding information, the tracking error increased with the velocity error increased. The tracking error increased to 22° when the velocity error was 1.7 m/s.

**Figure 5:**
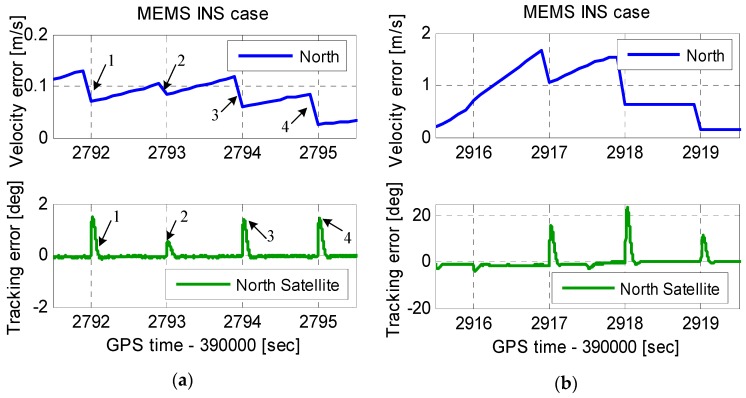
Zoomed-in INS velocity error and PLL tracking error. (**a**) Zoomed-in error under low dynamics and (**b**) zoomed-in error under high dynamics.

To analyze the aiding information impact on the tracking error thoroughly, Figure 4 is zoomed in as Figure 5. The INS velocity error had a step change at each integer second. This was because the INS velocity error was modified by the coupled filter output at each integer second. The INS velocity error modification was helpful to the navigation results, but harmful to assist the PLL operation. The tracking error amplitude was proportional to the step change at each integer second, which could not be ignored even under low dynamics. Therefore, the step change can deteriorate the carrier phase accuracy, and even lead to loss of lock of carrier phase. This problem was only mentioned in the analysis to the impacts of IMU in deep integration by Niu et al. [26]. However, the solution of this problem was not yet reported to the best of the authors’ knowledge. The improved INS-aided PLL structure is proposed to eliminate the impact of the step change in next section.

## 3. Improvement of INS-Aided PLLs

**Figure 6:**
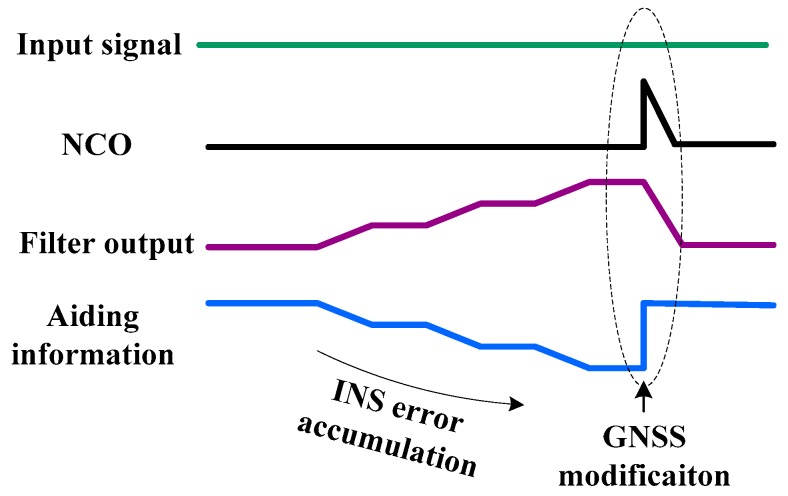
The principle of the step change impact on the PLL.

The testing results showed that the tracking error caused by the aiding information was proportional to the step change of the INS velocity error at each integer second. This phenomenon can be explained from the theory of the INS-aided PLL. Due to the inertial sensor error, the INS velocity error was diverging over time. And with the dynamics increasing, the INS velocity error caused by the inertial sensor error became larger. When the GNSS observations were updated at each integer second, the INS velocity error was corrected by the coupled filter output [26]. As we see in Figure 1, the PLL NCO input is the sum of the filter 2 output and the aiding information. While the aiding information provides the estimated Doppler between satellite and vehicle, the filter outputs the residuals of the Doppler estimation. Due to the INS error divergence before the GNSS updates, the aiding information error accumulates, shown in Figure 6. To keep the NCO synchronous to the input signal, the filter would output a value to cancel the aiding information error. That means the change of the filter output is opposite to the aiding information error. When the GNSS data updated at each integer second, the INS error was modified. Hence, the aiding information appeared as a step change, sent to the NCO. But the filter could not percept the step change before the loop updates. Therefore, the step change in the NCO caused the tracking error increase, which was consistent with the testing results.

**Figure 7:**
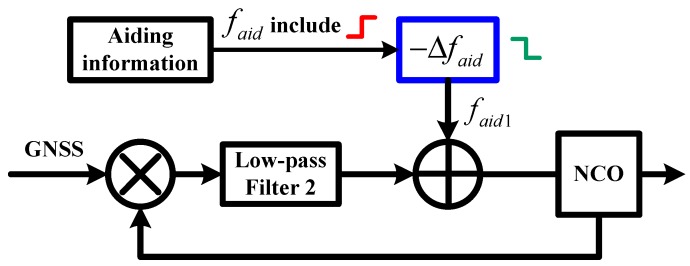
Improved structure of INS-aided PLL.

The improved structure of INS-aided PLLs is shown in Figure 7. There is a frequency step $\Delta f_{\mathrm{aid}}$ in the aiding information $\Delta f_{\mathrm{aid}}$ at each integer second, caused by the modification of INS velocity. To eliminate the impact of the frequency step on PLL tracking, $\Delta f_{\mathrm{aid}}$ was compensated before the aiding information is sent into the NCO of the PLL. Since the modification of INS velocity is output from the coupled Kalman filter, we can obtain the negative value of the frequency step. Therefore, a negative frequency step $-\Delta f_{\mathrm{aid}}$ was compensated to the aiding information to eliminate the impact of the frequency step.

**Figure 8:**
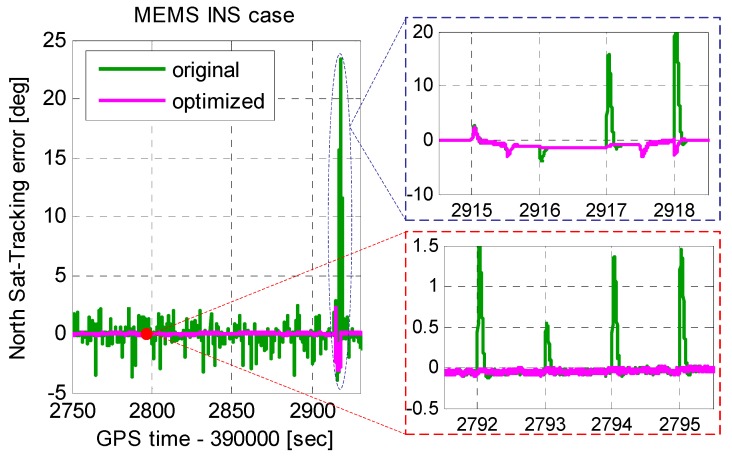
Tracking errors of improved INS-aided PLL.

Improved MEMS INS-aided PLLs were realized in our deeply-coupled system and were tested using the scenario in Section 2. Figure 8 shows the tracking errors of original INS-aided PLL and improved INS-aided PLL. Compared to the original INS-aided PLL, the tracking error caused by the INS velocity step was eliminated in the improved INS-aided PLL. Under low dynamics, the tracking error caused by the optimized aiding information was almost equal to zero, which can be ignored. Even when the acceleration was 1000 m/s^2^, the tracking error caused by the optimized aiding information was no more than 3°. Therefore, with the MEMS INS aiding, the PLL bandwidth did not need to be widened, and the carrier phase error had little increase under high dynamics.

## 4. Testing and Verification

To improve the carrier phase accuracy under high dynamics, we improved INS-aided PLLs and analyzed the tracking error by simulation. To evaluate INS-aided PLL performance, our deeply-coupled system was tested under the field condition. The tracking and navigation results of stand-alone PLLs and MEMS INS-aided PLLs were compared.

### 4.1. Description of the Test Method

**Figure 9:**
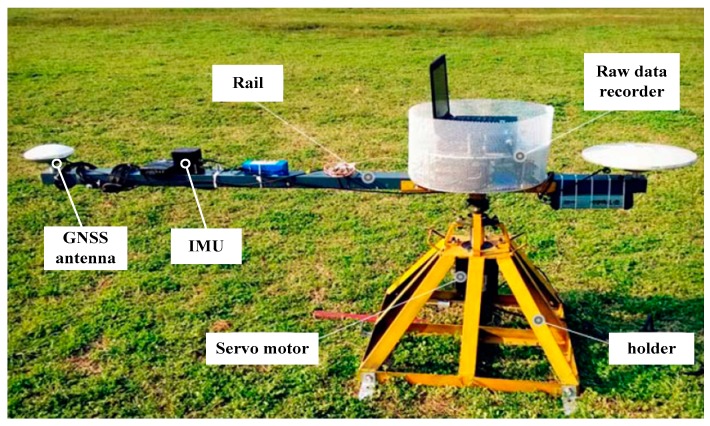
A spinning platform used to carry out the dynamic test.

A spinning platform, which can have a high-speed circular movement, was used to carry out the dynamic test, shown in Figure 9. The speed of the servo motor could be set by control software. The GNSS antenna, the MEMS IMU STIM300, and the raw data recorder were fixed on the rail. The turning radius of the GNSS antenna was 1.5 m, and that of the IMU was 1.0 m. When the rail spun centering on the servo motor, the antenna and the IMU moved with large acceleration.

**Figure 10:**
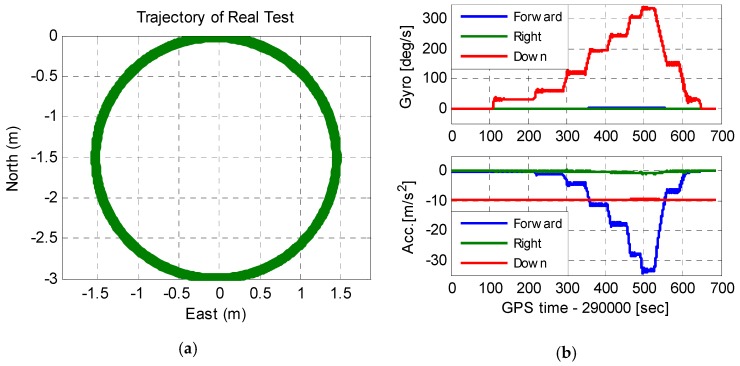
Antenna movement trajectory and the IMU raw output. (**a**) Antenna movement trajectory and (**b**) the IMU raw output.

Figure 10 shows the antenna movement trajectory and the IMU raw output. As seen in Figure 10a, the antenna had a circular motion with a radius of 1.5 m. The output of the IMU showed that the test platform started to accelerate at the 100th second, and decelerate gradually when the velocity was large enough. The maximum acceleration of the IMU was 34.5 m/s^2^, and the maximum angular velocity was 340°/s. Since the turning radius of the GNSS antenna was 1.5 times of that of the IMU, the maximum acceleration of the antenna was 51.75 m/s^2^, and the maximum jerk of the antenna was larger than 300 m/s^3^.

### 4.2. Signal Tracking Results Comparison

Taking satellite No.14 with an elevation of 60°, and satellite No.26 with an elevation of 30° as examples, tracking errors of second-order PLLs, third-order PLLs, and MEMS INS-aided second-order PLLs were tested.

**Figure 11:**
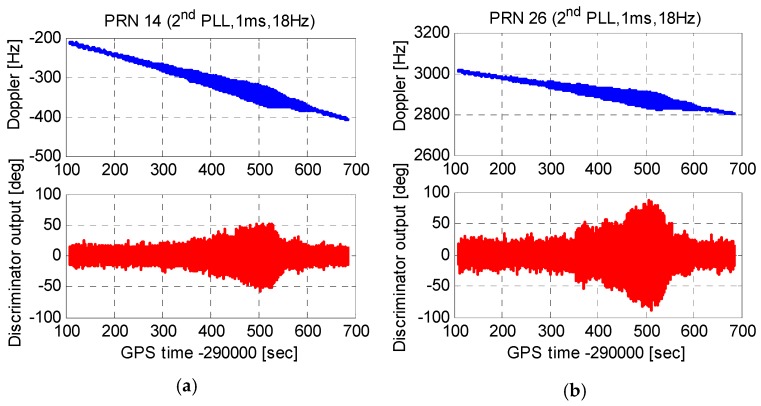
Carrier phase tracking errors of second-order PLLs. (**a**) Tracking errors of satellite No.14 and (**b**) tracking errors of satellite No.26.

Figure 11 shows carrier phase tracking errors of second-order PLLs with an integration time of 1 ms and a bandwidth of 18 Hz. With the increase of the platform acceleration, tracking errors enlarged obviously. The maximum tracking errors of satellite No.14 and satellite No.26 both exceeded the tracking threshold of 45°. That is, the second-order PLLs lost lock under the maximum platform acceleration. In addition, the tracking error of the satellite No.26 was larger than that of the satellite No.14. This is because, compared to the high elevation satellite, the line of sight (LOS) acceleration between the antenna and the low elevation satellite was larger.

**Figure 12:**
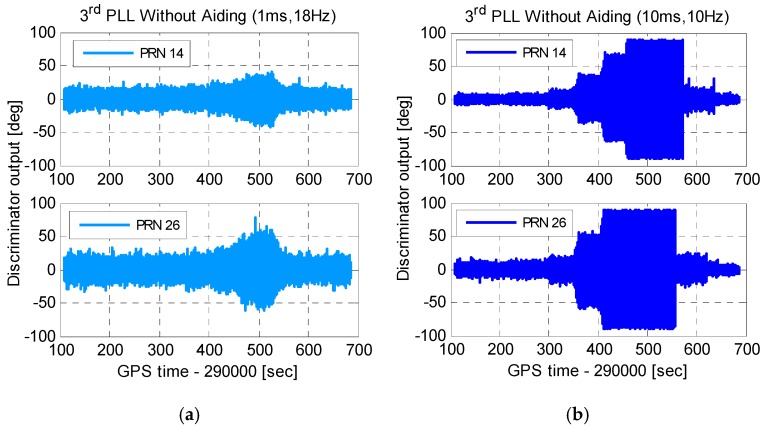
Carrier phase tracking errors of third-order PLLs. (**a**) PLL with an integration time of 1 ms and a bandwidth of 18 Hz and (**b**) PLL with an integration time of 10ms and a bandwidth of 10 Hz.

Figure 12 shows carrier phase tracking errors of third-order PLLs. Comparing Figure 12a to Figure 11, with the same integration time and bandwidth, carrier phase tracking errors of third-order PLLs were smaller than that of second-order PLLs under dynamics. To reduce the tracking error caused by thermal noise, the integration time should be lengthened, and the bandwidth should be narrowed. Tracking errors of third-order PLLs with an integration time of 10 ms and a bandwidth of 10 Hz are shown in Figure 12b. Compared to Figure 12a, though tracking errors reduced under statics, tracking errors increased greatly, and caused PLLs to lose lock under dynamics. Therefore, to tolerate dynamic stress, stand-alone PLLs should use short integration time and wide bandwidth, which increases carrier phase noise, especially for weak GNSS signals.

**Figure 13:**
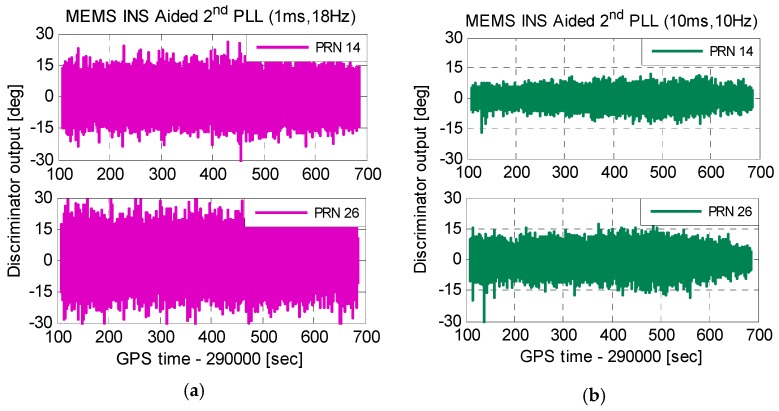
Carrier phase tracking errors of improved INS-aided second-order PLLs. (**a**) PLL with an integration time of 1 ms and a bandwidth of 18 Hz and (**b**) PLL with an integration time of 10 ms and a bandwidth of 10 Hz.

Figure 13 shows carrier phase tracking errors of improved INS-aided second-order PLLs. As is seen in Figure 13a, with the Doppler aiding of a MEMS INS, tracking errors of second-order PLLs, with an integration time of 1 ms and a bandwidth of 18 Hz, had no increase under dynamics. This illustrates that INS aiding could reduce the impact of dynamics on PLLs. The test results of MEMS INS-aided second-order PLLs with an integration time of 10 ms and a bandwidth of 10 Hz are shown in Figure 13b. On the one hand, tracking errors caused by thermal noise were suppressed. On the other hand, tracking errors did not increase obviously under dynamics. The results are consistent with the simulation. Therefore, the improved MEMS INS-aided PLLs can suppress tracking errors by thermal noise and dynamics simultaneously, and improve carrier phase measurement accuracy under dynamic environments.

### 4.3. Navigation Results Comparison

**Figure 14:**
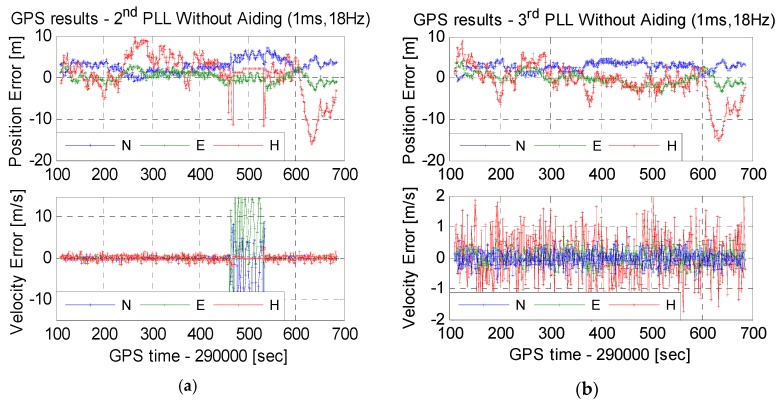

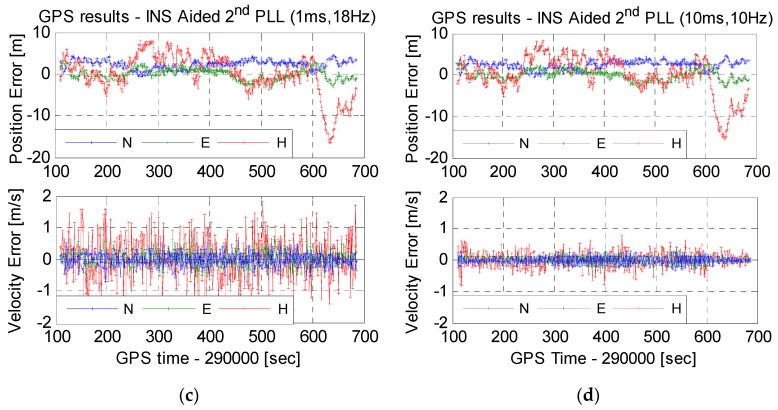
Position and velocity errors based on different PLLs. (**a**) Stand-alone second-order PLLs, (**b**) stand-alone third-order PLLs, (**c**) INS aided PLL with an integration time of 1 ms and a bandwidth of 18 Hz and (**d**) INS aided PLL with an integration time of 1 ms and a bandwidth of 18 Hz.

The test results in Section 4.2 show that stand-alone PLLs should use short integration time and wide bandwidth to keep lock under dynamics. Hence, position and velocity results are compared, shown in Figure 14, based on stand-alone second-order PLLs with an integration time of 1 ms and a bandwidth of 18 Hz, third-order PLLs with an integration time of 1 ms and a bandwidth of 18 Hz, MEMS INS-aided second-order PLLs with an integration time of 1 ms and a bandwidth of 18 Hz, and MEMS INS-aided second-order PLLs with an integration time of 10 ms and a bandwidth of 10 Hz. The position errors based on the four kinds of PLLs did not have significant differences. This is because the position results were calculated by pseudo-ranges, generated by code tracking loops, not obviously affected by PLLs, except for losing lock.

Velocity results were calculated by Doppler, generated by PLLs. As is shown in Figure 14a, when the platform acceleration increased to the maximum, the velocity error was extremely large. This was caused by losing lock of second-order PLLs. The velocity error in Figure 14b did not increase obviously under dynamics. This is because a phase error of 50° equates to a Doppler error of 0.14 Hz, only leading to a velocity error of 0.03 m/s. If the carrier phase was used for positioning, the position error would increase obviously under dynamics. Since stand-alone PLLs use short integration times and wide bandwidths under dynamics, the velocity noise was large. The root mean square (RMS) of horizontal velocity error was about 0.25 m/s, and the RMS of vertical velocity error was about 0.70 m/s. The velocity errors were compared, based on MEMS INS-aided second-order PLLs, with an integration time of 1 ms and a bandwidth of 18 Hz, and that with an integration time of 10 ms and a bandwidth of 10 Hz, shown in Figure 14c,d, respectively. When the integration time was lengthened and the bandwidth was narrowed, the velocity noise reduced obviously. The RMS of horizontal velocity error was about 0.11 m/s, and the RMS of vertical velocity error was about 0.20 m/s. Therefore, the velocity accuracy can be improved by MEMS INS-aided PLLs. The position based carrier phase may be realized through testing in future work.

## 5. Conclusions

To obtain accurate carrier phase for GNSS precision positioning under dynamics, we focused on improving the design of MEMS INS-aided PLLs. Typical MEMS INS-aided PLLs were implemented in our software GNSS/INS deeply-coupled system, and tracking errors caused by aiding information were tested and analyzed under a jerk of 2000 m/s^3^ using hardware simulator. Results showed that there was a step change in the aiding information at each integer second, which deteriorated the carrier phase accuracy, even leading to the unlocked PLL. The improved structure of MEMS INS-aided PLLs is proposed to eliminate the step change impact on tracking error. Results showed that even when the jerk was 2000 m/s^3^, the tracking error caused by the optimized aiding information was no more than 3°. Finally, the tracking and navigation performances of stand-alone PLLs and INS-aided PLLs were tested using a spinning platform under the field condition. The stand-alone PLLs could not lengthen integration time and reduce bandwidth to suppress noise, and the carrier phases significantly increased under dynamics. The improved MEMS INS-aided PLLs can suppress tracking errors caused by thermal noise and dynamics simultaneously, and improve carrier phase measurement accuracy under dynamic environments.

## References

[1] Puente I., Gonzalez Jorge H., Martinez Sanchez J., Arias P. (2013). Review of mobile mapping and surveying technologies. Measurement.

[2] Luettel T., Himmelsbach M., Wuensche H. (2012). Autonomous ground vehicles—Concepts and a path to the future. IEEE Proc..

[3] Bock Y., Prawirodirdjo L., Melbourne T.I. (2004). Detection of arbitrarily large dynamic ground motions with a dense high-rate GPS network. Geophys. Res. Lett..

[4] Pownell M., Nielson J., Madsen J. Use of GPS for long range precision navigation for weapon delivery. Proceedings of the ION GNSS.

[5] Kaplan E., Hegarty C. (2006). Understanding GPS: Principles and Applications.

[6] Jwo D.J. (2001). Optimization and sensitivity analysis of GPS receiver tracking loops in dynamic environments. IEEE Proc. Radar Sonar Navig..

[7] Moschas F., Stiros S. (2015). PLL bandwidth and noise in 100 Hz GPS measurements. GPS Solut..

[8] Ebinuma T., Kato T. (2012). Dynamic characteristics of very-high-rate GPS observations for seismology. Earth Planets Space.

[9] Misra P., Enge P. (2006). Global Positioning System: Signals, Measurements, and Performance.

[10] Niu X., Zhang Q., Gong L., Liu C., Zhang H., Shi C., Wang J., Coleman M. (2015). Development and evaluation of GNSS/INS data processing software for position and orientation systems. Surv. Rev..

[11] Kennedy S., Hamilton J., Martell H. Architecture and System Performance of SPAN-NovAtel’s GPS/INS Solution. Proceedings of the IEEE/ION PLANS.

[12] Petritoli E., Giagnacovo T., Leccese F. Lightweight GPS/IRS AIME integrated navigation system for UAV vehicles. Proceedings of the IEEE International Workshop on Metrology for Aerospace.

[13] Petritoli E., Leccese F. Improvement of altitude precision in indoor and urban canyon navigation for small flying vehicles. Proceedings of the 2nd IEEE International Workshop on Metrology for Aerospace.

[14] Eling C., Klingbeil L., Kuhlmann H. (2015). Real-time single-frequency GPS/MEMS-IMU attitude determination of lightweight UAVs. Sensors.

[15] Gautier J.D., Parkinson B.W. Using the GPS/INS generalized evaluation tool (GIGET) for the comparison of loosely coupled, tightly coupled and ultra-tightly coupled integrated navigation systems. Proceedings of the ION AM.

[16] Gao G., Lachapelle G. INS-assisted high sensitivity GPS receivers for degraded signal navigation. Proceedings of the Institute of Navigation.

[17] Tsujii T., Fujiwara T., Kubota T., Kubo Y. (2014). Testing of an ultra-tightly coupled GPS/INS under strong ionospheric scintillation. ISCIE.

[18] Alban S., Akos D., Rock S. Performance analysis and architectures for INS-aided GPS tracking loops. Proceedings of the Institute of Navigation National Technical Meeting.

[19] Alban S. (2004). Design and Performance of a Robust GPS/INS Attitude System for Automobile Applications.

[20] Gebre-Egziabher D., Razavi A., Enge P., Gautier J., Pullen S., Pervan B.S., Akos D.M. (2005). Sensitivity and performance analysis of doppler-aided GPS carrier-tracking loops. J. Inst. Navig..

[21] Chiou T.Y. GPS receiver performance using inertial-aided carrier tracking loop. Proceedings of the 18th International Technical Meeting of the Satellite Division of the Institute of Navigation.

[22] Yang Y., EI-Sheimy N. Improving GPS receiver tracking performance of PLL by MEMS IMU aiding. Proceedings of the ION GNSS.

[23] Zhang T., Zhang H., Ban Y., Yan Y., Niu X., Liu J. (2013). Hardware implementation of a real-time MEMS IMU/GNSS deeply-coupled system. IEICE Trans. Commun..

[24] Irsigler M., Eissfeller B. (2002). PLL tracking performance in the presence of oscillator phase noise. GPS Solut..

[25] Sensonor STIM300. http://www.sensonor.com/gyro-products/inertial-measurement-units/stim300.aspx.

[26] Niu X., Ban Y., Zhang Q., Zhang T., Zhang H., Liu J. (2015). Quantitative analysis to the impacts of IMU quality in GPS/INS deep integration. Micromachines.

